# Protective Effect of Water-Soluble C_60_ Fullerene Nanoparticles on the Ischemia-Reperfusion Injury of the Muscle Soleus in Rats

**DOI:** 10.3390/ijms22136812

**Published:** 2021-06-24

**Authors:** Dmytro Nozdrenko, Tetiana Matvienko, Oksana Vygovska, Kateryna Bogutska, Olexandr Motuziuk, Natalia Nurishchenko, Yuriy Prylutskyy, Peter Scharff, Uwe Ritter

**Affiliations:** 1 Department of Biophysics and Medical Informatics, Taras Shevchenko National University of Kyiv, 01601 Kyiv, Ukraine; ddd@univ.kiev.ua (D.N.); tamatvienko@gmail.com (T.M.); bogutska_ki@knu.ua (K.B.); natnurish@gmail.com (N.N.); prylut@ukr.net (Y.P.); 2 Department of Biology, Bogomolets National Medical University of Kyiv, 01601 Kyiv, Ukraine; ovvigovskaya@gmail.com; 3 Department of Human and Animal Physiology, Lesya Ukrainka Volyn National University, 43025 Lutsk, Ukraine; cmoplutsk@gmail.com; 4 Institute of Chemistry and Biotechnology, Technical University of Ilmenau, 98693 Ilmenau, Germany; peter.scharff@tu-ilmenau.de

**Keywords:** C_60_ fullerene, muscle soleus of rat, ischemia, biomechanical and biochemical parameters

## Abstract

The biomechanical parameters of muscle soleus contraction in rats and their blood biochemical indicators after the intramuscular administration of water-soluble C_60_ fullerene at doses of 0.5, 1, and 2 mg/kg 1 h before the onset of muscle ischemia were investigated. In particular, changes in the contraction force of the ischemic muscle soleus, the integrated power of the muscle, the time to achieve the maximum force response, the dynamics of fatigue processes, and the parameters of the transition from dentate to smooth tetanus, levels of creatinine, creatine kinase, lactate and lactate dehydrogenase, and parameters of prooxidant–antioxidant balance (thiobarbituric acid reactive substances, hydrogen peroxide, and reduced glutathione and catalase) were analyzed. The positive therapeutic changes in the studied biomechanical and biochemical markers were revealed, which indicate the possibility of using water-soluble C_60_ fullerenes as effective prophylactic nanoagents to reduce the severity of pathological conditions of the muscular system caused by ischemic damage to skeletal muscles.

## 1. Introduction

Among the muscle pathologies that develop in skeletal muscles in various injuries, ischemic injuries account for more than 35% of the total number of injuries to the musculoskeletal system. Ischemic reperfusion injuries of skeletal muscles after acute arterial occlusion, in many cases, are the cause of severe pathologies and mortality [1]. Ischemic tissue damage is a cascade of biochemical reactions that are initiated under conditions of hypoxia after a few minutes of ischemia as a result of insufficient blood supply [2]. The ischemic cascade usually continues for 2–3 h after ischemia, but can last for several days, even after normal blood flow has been restored [3]. At the same time, with ischemia lasting 3 h or more, both muscle necrotic changes and nervous degradation occur. The amount of necrosis in the muscle tissue can be up to 60% [4]. In addition, with ischemic reperfusion, the expression of adhesive molecules on the endothelium is increased. Activated neutrophils attracted to the site of injury release free radicals [2]. The last ones provoke vasoconstriction, which is a characteristic manifestation of ischemic damage. In addition, ischemia−reperfusion injury of skeletal muscles is one of the main causes of post-traumatic pathologies after surgical procedures [5,6]. The main goal in the treatment of muscle ischemia is the rapid restoration of blood flow in the damaged areas. However, such therapy often leads to a new pathophysiological process-reperfusion injury, which can also cause significant damage to the muscle tissue. The rapid establishment of the severity of ischemic injury is critical for further therapy; however, there are currently no accurate diagnostic tests to achieve this goal [7]. Literature data indicate that during reperfusion, free radicals, together with calcium activated caspases and calpains, can lead to apoptosis and damage to the DNA and mitochondria, resulting in additional loss of muscle functions [8,9]. So, the interaction of the hydroxyl radical with the hydrogen atoms of the methyl groups of polyunsaturated fatty acids initiates the peroxidation of the membrane lipids, which in turn leads to increased permeability of the cell membranes [2].

It is known that C_60_ fullerenes efficiently capture and inhibit free radicals in in vivo and in vitro systems [10,11,12]. Whether the double chemical bonds in the structure of C_60_ fullerene are electron-deficient determines its ability to attach up to six electrons [13]. In our previous work, it was shown that the administration of biocompatible water-soluble C_60_ fullerenes [14] after the initiation of ischemic damage to the skeletal muscle leads to a significant positive therapeutic effect [15]. At the same time, it was revealed that the administration of C_60_ fullerenes directly into the damaged muscle complicates their steady distribution over the tissues and, thus, reduces the antioxidant effect of the drug. In this case, the time elapsed after the initiation of ischemia before the administration of the therapeutic drug is of great importance, as the beginning of the ischemic cascade of muscle tissue damage occurs already in the first seconds after reperfusion [16]. All of this served as the basis for further investigation of the effect of C_60_ fullerene aqueous solution (C_60_FAS) on the dynamics of the contractile process of muscle soleus in rats against the background of ischemic pathology when administered intramuscularly 1 h before the initiation of ischemia, depending on the dose (protective effect).

## 2. Results and Discussion

### 2.1. Characterization of C_60_FAS

The monitoring of the C_60_ fullerene morphology in an aqueous solution is important for controlling the particle size distribution profile, which may influence the C_60_FAS bioactivity and toxicity [17,18,19,20]. The prepared C_60_FAS was characterized by atomic force microscopy (AFM) and scanning tunneling microscopy (STM).

**Figure 1:**
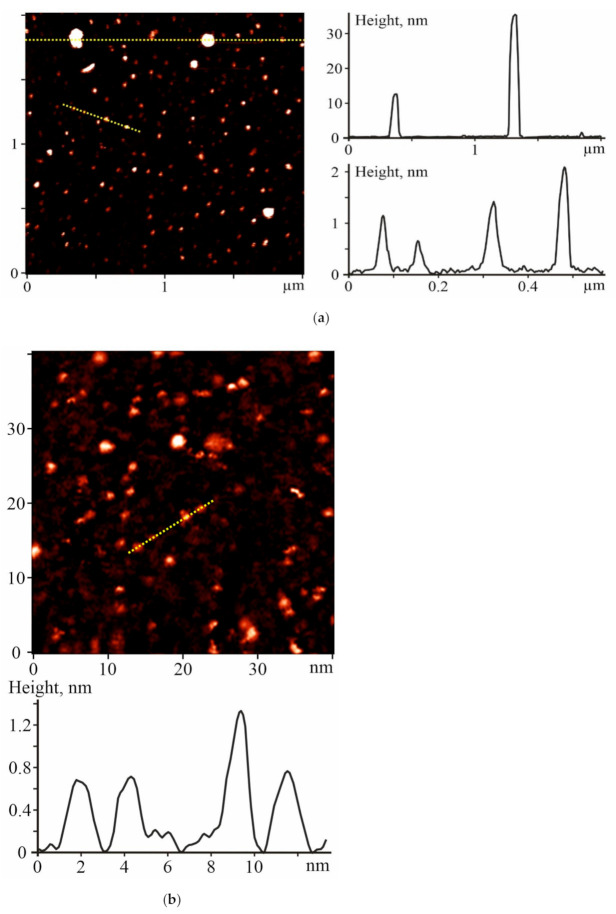
(**a**) Atomic force microscopy (AFM) and (**b**) scanning tunneling microscopy (STM) images of the C_60_ fullerene nanoparticles on the mica and gold surfaces, respectively, and their profiles along the marked lines. C_60_ fullerenes were precipitated from C_60_FAS with a 0.15 mg/mL concentration.

The study of the C_60_ fullerene films deposited from an aqueous solution revealed a high degree of molecule dispersion in the solution. It turned out that the prepared C_60_FAS contained both single C_60_ fullerene and its labile nanoaggregates with a size of 1.3–35 nm. The majority of C_60_ molecules were located chaotically and separately along the surface (see the objects with a height of ~0.7 nm in Figure 1), or in the form of bulk clusters consisting of several tens of C_60_ molecules [21] (objects with a height of 1.3–2 nm in Figure 1. Such an arrangement of C_60_ molecules formed because of electrostatic repulsion between them; the zeta potential value was −25.3 mV at room temperature [22], indicating a high solute stabilization.

### 2.2. Biomechanics of Injured Muscle Contractions

**Figure 2:**
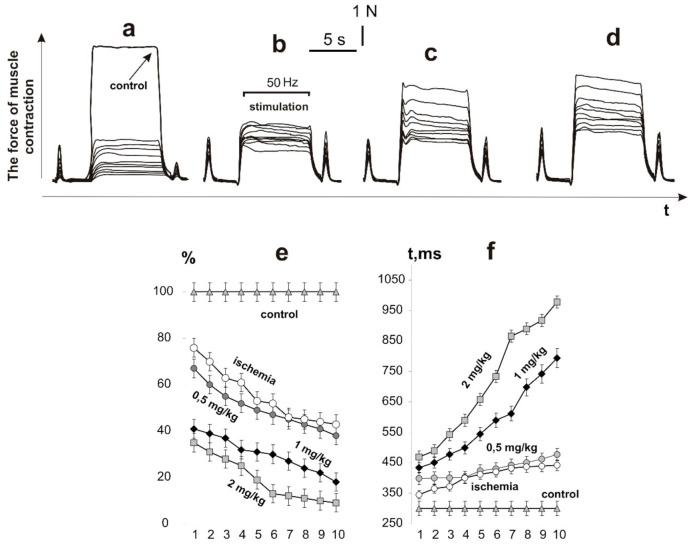
The force of contraction of the rat muscle soleus, caused by 10 (indicated 1,2, …, 10) consecutive 6 s non-relaxation pools of stimulation: ischemic muscle without C_60_FAS (control: native muscle) (**a**); administration of C_60_FAS 1 h before muscle ischemia at doses of 0.5 (**b**), 1 (**c**), and 2 mg/kg (**d**). Integrated muscle power, calculated area under the force curve, as a percentage of control values (**e**). Time to reach the maximum force response (**f**).

After the initiation of ischemic damage, the contraction force of the rat muscle soleus, caused by 6 s non-relaxation stimulation pools, decreased to 28 ± 2% of the control values at the first contraction and to 9 ± 1% at the tenth (Figure 2). The decrease in the integrated power of the muscle contraction was 39 ± 2% of the control values at the first contraction and 6 ± 2% at the tenth, respectively. The time to reach the maximum force response increased from 451 ± 5 ms at the first contraction to 978 ± 7 ms at the tenth. Thus, a sharp decrease in the force activity of the muscle was observed at the first contractions with a progressive decrease in biomechanical parameters. This confirms the literature data that in the process of ischemia−reperfusion, a significant decrease in the force of the contraction of skeletal muscle occurs. The progressive decrease in the force response lasts at least 5 days, after which the recovery process takes place [23,24].

The use of C_60_FAS injections increased the muscle force response as follows: at a dose of 0.5 mg/kg of C_60_FAS, 58 ± 1% and 51 ± 1% of the control values on the first and tenth contractions, respectively; at a dose of 1 mg/kg of C_60_FAS, 78 ± 2% and 56 ± 2%, respectively; and at a dose of 2 mg/kg of C_60_FAS, 79 ± 1% and 58 ± 1%, respectively. At a dose of 0.5 mg/kg of C_60_FAS, the integrated power of the muscle contraction was 54 ± 2% of the control values at the first contraction and 52 ± 2% at the tenth, respectively. After increasing the doses of C_60_FAS, this parameter was 76 ± 1% and 55 ± 1% at 1 mg/kg and 78 ± 2% and 59 ± 2% at 2 mg/kg, respectively. The time to reach the maximum force response increased from 373 ± 3 ms at the first contraction to 755 ± 6 ms at the tenth at a dose of 0.5 mg/kg C_60_FAS; from 343 ± 4 ms at the first contraction to 457 ± 6 ms at the tenth at a dose of 1 mg/kg of C_60_FAS; and from 291 ± 5 ms at the first contraction to 399 ± 7 ms at the tenth at a dose of 2 mg/kg of C_60_FAS.

It is important to note that after the administration of C_60_FAS, the force response of the ischemic muscle did not decrease by more than 50% of the control values, even with the tenth act of contraction. At the same time, the C_60_FAS dose increasing from 1 to 2 mg/kg did not lead to significant therapeutic effects. Thus, the data obtained indicate a significant positive trend in the use of C_60_FAS for prophylactic purposes. Based on the data obtained, it can be concluded that pretraumatic administration of C_60_FAS at a dose of 1 mg/kg reduces the severity of ischemic damage in the muscle by 60–75%. A decrease in the C_60_FAS dose leads to a decrease in the therapeutic effect, while its increase does not lead to a significant increase in the biomechanical parameters. In addition, it should be noted that the use of C_60_ fullerene therapy did not eliminate the developing fatigue processes in the ischemic muscle; the integrated muscle power decreased with each subsequent pool of the stimulation signal. Therefore, the next stage of the study was to investigate the nature of the muscle response during prolonged fatigue stimulation. At this stage, we applied only one, the most optimal, dose of C_60_FAS of 1 mg/kg.

**Figure 3:**
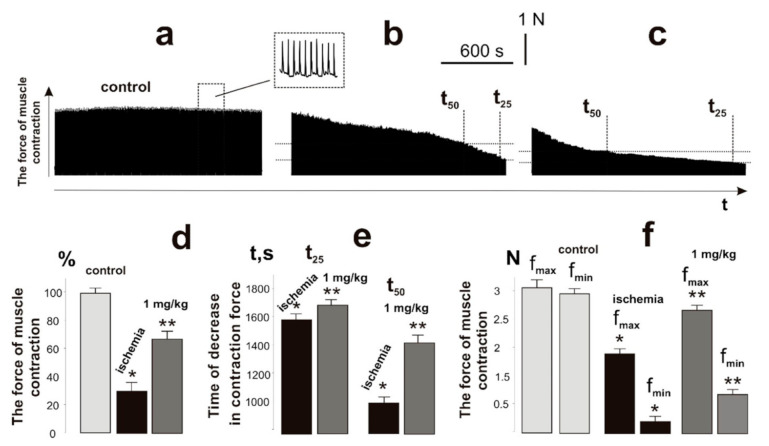
Registration of the force of contraction of the muscle soleus of the rat with the use of 1 Hz stimulation with a duration of 1800 s: control, native muscle (**a**); ischemic muscle without the administration of C_60_FAS (**b**); administration of C_60_FAS (1 mg/kg) 1 h before muscle ischemia (**c**); integrated muscle power, presented as a percentage of the control values (**d**); the time of the decrease in the force response by 50% and 25% of the initial values (t_50_ and t_25_) (**e**); and maximum (f_max_) and minimum (f_min_) fixed forces of muscle contraction throughout the entire duration of stimulation (**f**). * *p* < 0.05 relative to the control group; ** *p* < 0.05 relative to the ischemia group.

It has been shown that ischemia−reperfusion increases the degree of fatigue processes development and reduces the force of muscle contraction to 40% after 1 h of ischemia and to 70% after 3 h. Recovery of the muscle force response was observed only at the end of the second week after ischemia−reperfusion [6]. Registration of the contraction force of the ischemic muscle soleus of a rat with 1 Hz stimulation for 1800 s (Figure 3b,c) revealed a decrease in the integrated muscle power (Figure 3d), which was 35 ± 4% of the control value. Intramuscular injections of C_60_FAS changed this parameter to 67 ± 4%. The time for the decrease in the force response by 50% and 25% from the initial values was 940 ± 11 s and 1580 ± 18 s, respectively, without C_60_ fullerene therapy, and 1430 ± 17 s and 1690 ± 14 s, respectively, with the administration of C_60_FAS (Figure 3e). The maximum and minimum recorded contraction forces of the ischemic muscle throughout the entire duration of stimulation were 1.8 ± 0.3 N (3.1 ± 0.4 N in control) and 0.18 ± 0.01 N (2.9 ± 0.4 N in control), respectively (Figure 3f). When C_60_FAS was injected, this indicator was 2.5 ± 0.4 N and 0.6 ± 0.1 N, respectively, which shows its 52% therapeutic effect at the stages of maintaining maximum force responses during the development of fatigue processes.

In the process of skeletal muscle functioning, the most important quality indicator of its work is the rate of occurrence of smooth tetanic contraction (a state of continuous muscle tension after complete summation of single contractions). Even minimal changes in the structure of the impulses generated by motor neurons, damage to myocyte membranes, development of the inflammatory process, changes in muscle stiffness, electrical properties of membranes, and the duration of hyperpolarization significantly change the time of occurrence of smooth tetanic contractions [25,26]. In addition, during muscle activity, its individual motor units generate non-fused tetanic contractions, which are characterized by variable strength and varying degrees of fusion. The synchronization of this process depends on many factors and is also a vulnerable link in the development of pathological processes in the muscle [27,28]. Therefore, the next stage of the study was to investigate the biomechanical markers of the appearance of smooth tetanic contractions in the ischemic muscle soleus of the rat.

**Figure 4:**
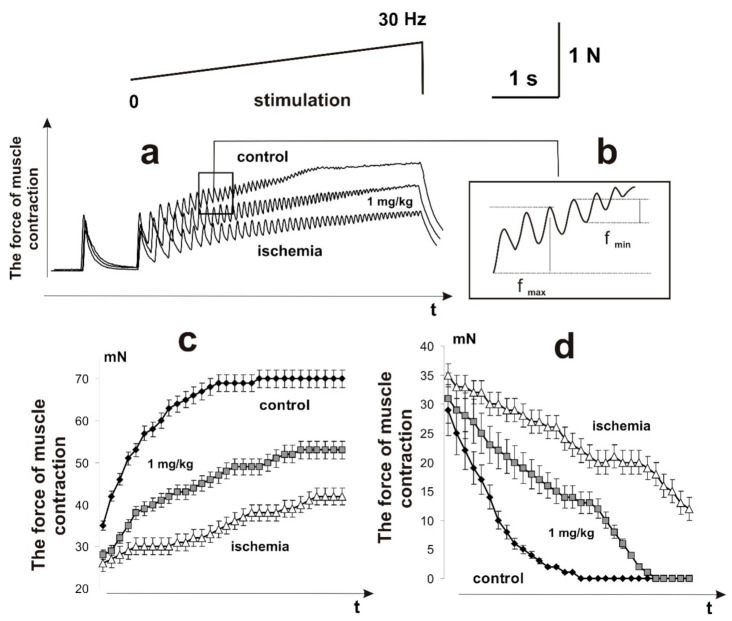
Biomechanical parameters of muscle soleus transition from dentate to smooth tetanus after using increasing stimulation with a maximum frequency of 30 Hz for 6 s: mechanograms of the native muscle contraction, control (**a**); f_max_ is the maximum force of a single contraction, f_min_ is the minimum value of the force response in one spike of the dentate tetanus (a decrease in this parameter to zero leads to the appearance of smooth tetanus) (**b**); and changes in the parameters f_max_ (**c**) and f_min_ (**d**) for each of the single contractions before the transition of the force response to smooth tetanus when an increasing stimulation signal is applied.

The smooth tetanic contractions (maximum force response) appeared in 3450 ± 12 ms and reached 70 ± 8 mN after using stimulation pools of increasing frequency (Figure 4). The ischemically damaged muscle throughout the entire stimulation pool did not reach the stage of smooth tetanic contraction. The maximum force of a single contraction increased from 24 ± 2 mN to 37 ± 3 mN. The minimum value of the force response in one spike of the dentate tetanus decreased to 12 ± 1 mN. It should be noted that a decrease in this parameter to zero leads to the appearance of smooth tetanus. Preliminary injections of C_60_FAS changed the biomechanical parameters of ischemized muscle soleus transition from dentate to smooth tetanus, which appeared after 4950 ± 32 ms and reached 58 ± 2 mN. It should be noted that the injection of C_60_FAS eliminated both the abrupt decrease in the force of contraction and the fluctuation component of the contractile process. Thus, the preventive effect of C_60_FAS injection on the biomechanical parameters of the transition of ischemic muscle from dentate to smooth tetanus was 68 ± 4% of the control values.

### 2.3. Blood Biochemical Indicators of Rats with Injured Muscle

**Figure 5:**
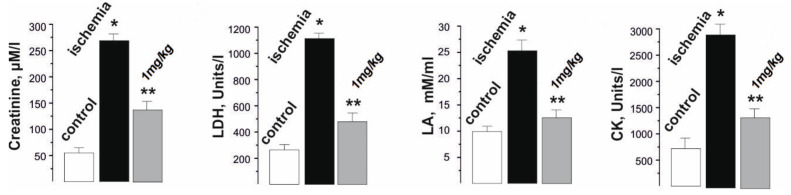
Biochemical indicators (creatinine, lactate dehydrogenase (LDH), lactate (LA), and creatine kinase (CK)) in the blood of rats after 1 Hz stimulation of the ischemic muscle soleus for 1800 s. * *p* < 0.05 relative to the control group; ** *p* < 0.05 relative to the ischemia group.

The analysis of the biochemical composition of the blood of rats during the development of ischemia−reperfusion reflected the changes occurring in the damaged skeletal muscle and made it possible to evaluate the therapeutic effect of the applied drug on the pathological process. The biochemical indicators of the development of fatigue processes selected by us for the study, such as creatinine, lactate dehydrogenase (LDH), lactate (LA), and creatine kinase (CK) are also indicators of physiological disorders in the muscle tissue due to the development of ischemic damage (Figure 5).

The change in the level of creatinine, a product formed in the muscles during the destruction of intramuscular structures, made it possible to assess the level of damage to the myocytes during prolonged contractions. This indicator increased from 50 ± 2 µM/L in the control to 25,750 ± 51 µM/L after muscle ischemia. The administration of C_60_FAS prior to muscle ischemia reduced this indicator to 122 ± 2 µM/L. In our opinion, the decrease in the creatinine fraction was due to the C_60_ molecules that protect the membranes of skeletal muscle cells from nonspecific free radical destruction, effectively absorbing the reactive oxygen species (ROS).

The level of changes in LDH, an enzyme that generates lactic acid, made it possible to assess the muscle performance after ischemia. The change in the level of this enzyme from 220 ± 8 units/l in the control to 1115 ± 22 units/l after ischemia is evidence of the development of significant muscle dysfunctions associated with the development of the inflammatory process. An increase in the LDH fraction in the blood is the result of both the physiological destruction of the myocyte walls caused by their performance [29] and an increase in LA content during prolonged muscle activation. Preliminary administration of C_60_FAS reduced the LDH level to 442 ± 11 units/l. A decrease in this enzyme upon the administration of C_60_FAS may indicate both a decrease in mechanical damage to muscle fibers and in LA concentration in the muscular system in general.

In active muscle, most metabolic and biochemical processes occur under anaerobic conditions; the muscle uses a significant amount of mitochondrial enzymes and, as a result, a large amount of LA accumulates in it, which cannot be oxidized during prolonged muscle stimulation. An increase in the level of lactic acid in active muscle indicates that the level of its entry into the cell exceeds the level of its oxidation and excretion. In the control values, the LA level was 11 ± 2 mM/mL. After ishimization, its value increased to 27 ± 3 mM/mL. C_60_FAS injections reduced the LA level to 17 ± 1 mM/mL. Thus, pre-C_60_ fullerene therapy led to a decrease in the LA level by almost 50%.

CK is an enzyme found in high concentrations in the skeletal muscle. The release of this enzyme from the cells and, accordingly, an increase in CK activity in the blood are observed after mechanical damage to the muscles. The increase in the CK fraction in the blood during the induction of ischemia from 560 ± 13 units/l in the control to 2830 ± 22 units/l is the result of the rapid physiological destruction of the myocyte walls, which intensifies during active prolonged non-relaxation muscle contraction. The CK level decreased significantly (more than three times) and reached 820 ± 23 units/l after the application of C_60_FAS. CK is an enzyme from the energy supply system of musculoskeletal cells that catalyzes the transfer of a phosphate group from ATP to a creatine molecule with the formation of a high-energy compound creatine phosphate, which is used by the body as an energy substance when physical activity increases. A change in its concentration is one of the known markers of the pathological processes in the muscle and characterizes the depletion of the cell’s energy reserves. So, it was shown that during 3 h of ischemia-reperfusion of muscle soleus the depletion of ATP reserves was about 95%, and glycogen was depleted by 88% [6]. From a functional point of view, these data indicate that a large amount of high-energy phosphate compounds is consumed by an ischemic-damaged muscle cell so as to maintain homeostasis and, as a consequence, metabolic disorders occur, leading to a significant increase in ischemic muscle fatigue. Thus, preliminary injections of C_60_FAS significantly increase the energy capabilities of actively contracting ischemic muscle.

**Figure 6:**
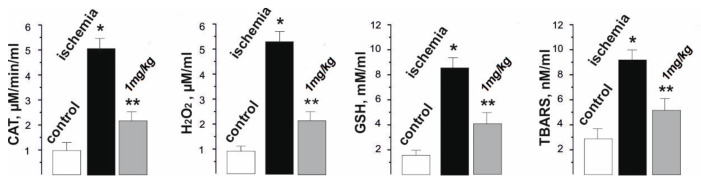
Levels of catalase (CAT), hydrogen peroxide (H_2_O_2_), and thiobarbituric acid reactive substances (TBARS) in rat blood after 1 Hz stimulation of the ischemic muscle soleus for 1800 s. * *p* < 0.05 relative to the control group; ** *p* < 0.05 relative to the ischemia group.

The pathological inflammatory processes that occur immediately after ischemia-reperfusion are a source of ROS and contribute to the intensification of lipid peroxidation processes [8]. This interferes with the adequate performance of muscle work and significantly increases the duration of the recovery period. During reperfusion, oxygen entering the tissues initiates the oxidation of xanthine and hypoxanthine by xanthine oxidase, which leads to the formation of a large amount of superoxide anion radical and hydrogen peroxide. Hydrogen peroxide is converted to hydroxyl radicals by the reduction of metal ions. Mitochondria damaged by ischemia can produce more electrons because of their “leakage” from the electron transport chain. These electrons are involved in the formation of the superoxide radical anion. In addition, during ischemia−reperfusion, the expression of adhesive molecules on the endothelium increases. Activated neutrophils attracted to the site of injury also release free radicals and provoke vasoconstriction, which is a characteristic manifestation of ischemic damage [2,3,4,5]. As a result of biochemical tests, the increased level of peroxidation markers and oxidative stress (catalase (CAT), hydrogen peroxide (H_2_O_2_), and thiobarbituric acid reactive substances (TBARS), and the reduced glutathione (GSH)) after muscle ischemia, as well as their significant decrease after C_60_FAS injections before muscle ischemia (Figure 6), were revealed.

So, the CAT activity increased from 0.9 ± 0.1 µM/min/mL in the control to 5.1 ± 0.3 µM/min/mL after muscle ischemia, and decreased to 2.1 ± 0.1 µM/min/mL with C_60_ fullerene therapy. The H_2_O_2_ level was 5.4 ± 0.4 µM/mL during ischemia (0.8 ± 0.1 µM/mL in the control) and 2.2 ± 0.2 µM/mL after the administration of C_60_FAS. The GSH concentration was 8.3 ± 0.6 mM/mL with ischemia (1.8 ± 0.1 mM/mL in the control) and 3.9 ± 0.2 mM/mL with C_60_FAS injection. Finally, the TBARS level was 9.8 ± 1.0 nM/mL with ischemia (2.3 ± 0.2 nM/mL in the control) and 5.8 ± 0.5 nM/mL with the administration of C_60_FAS.

Thus, there is a clear tendency towards a decrease in the described biochemical parameters by about 45–60% with the prophylactic use of C_60_FAS. We suppose that C_60_ fullerenes can affect the activity of endogenous antioxidants, preventing the onset of dysfunction in the active muscle and, thus, maintaining it within the physiological norm during the entire process of its contraction.

In summary, oxidative stress causes cellular damage in ischemic pathology. The mediators of oxidative stress are ROS, including superoxide anion radical, hydroxyl radical, singlet oxygen, and hydrogen peroxide, which damage cellular targets such as DNA, proteins, and lipids [30]. After ischemia, a sequential chain of pathophysiological cascades occurs, including massive intracellular release of Ca^2+^, disruption of the mitochondrial electron transport chain, release of neutrophils, acute inflammatory reactions, and the formation of free radicals, which, in turn, enhance apoptotic or necrotic cell death. The endogenous antioxidant defense system of the body, at the beginning of the development of the ischemic cascade, can neutralize only a small amount of ROS by enzymatic and non-enzymatic pathways [31].

The chemical structure of C_60_ fullerene with an abundance of conjugated double bonds and low-lying lower unoccupied molecular orbitals makes it very susceptible to free radicals. Thanks to this, C_60_ fullerene can react with many ROS without losing its antioxidant properties [32]. The protective effect of C_60_ fullerene on the absorption of superoxide anions does not lead to an increased production of hydrogen peroxide [33]. C_60_ fullerene promotes cell survival by altering the cellular redox state and enzyme activity [34]. C_60_ fullerene reduces lipid peroxidation by actively absorbing ROS [35]. C_60_ fullerenes can penetrate the cell membrane and localize in the mitochondria [36,37], which are the source of ROS during the development of ischemic cell damage. Finally, the obtained above results are also confirmed by the previously obtained data on the effect of water-soluble C_60_ fullerenes on the functions of the antioxidant systems of the body in inflammatory and pathological processes [38,39,40,41]. They indicate that the development of medical nanotechnology based on water-soluble C_60_ fullerenes, considering their powerful antioxidant properties, opens up new possibilities in the treatment and prevention of ischemic damage to skeletal muscles.

## 3. Materials and Methods

To obtain C_60_FAS, a method was used that is based on the transfer of C_60_ molecules from toluene to water, followed by sonication [42,43]. Briefly, a saturated solution of pure C_60_ fullerene in toluene (purity >99.5%), where its concentration corresponds to a maximum solubility of ~2.9 mg/mL, was mixed with the same volume of distillate in an open beaker. The formed aqueous phases was subjected to ultrasound (frequency 8 Hz, duration 8 h). The obtained C_60_FAS at the maximum concentration of C_60_ fullerene 0.15 mg/mL remained stable for 18 months at a temperature of +4 °C.

AFM and STM were performed to determine the size of the C_60_ fullerene particles in aqueous solution. Measurements were done with the “Solver Pro M” system (NT-MDT, Moscow, Russia). A drop of investigated solution was transferred on the atomic-smooth substrate to deposit layers. Measurements were carried out after complete evaporation of the solvent. For AFM studies, a freshly broken surface of mica (SPI supplies, V-1 grade) was used as a substrate. Measurements were carried out in a semicontact (tapping) mode with AFM probes of the RTPESPA150 (Bruker, Billerica, MA, 6 N/m, 150 kHz) type. STM studies were performed with the Au (111) surface obtained after annealing the substrates of Au/mica (Phasis, Switzerland) in a gas burner flame (propane−butane). The typical tunneling current and voltage values were 0.027–0.1 nA and 0.1–1 V, respectively.

The experiments were performed on male Wistar rats aged 3 months, weighing 170 ± 5 g. The study protocol was approved by the bioethics committee of ESC “Institute of Biology and Medicine”, Taras Shevchenko National University of Kyiv, in accordance with the rules of the European Convention for the Protection of Vertebrate Animals Used for Experimental and Other Scientific Purposes, and the norms of biomedical ethics in accordance with the Law of Ukraine № 3446—IV 21.02.2006, Kyiv, on the Protection of Animals from Cruelty during medical and biological research.

Fifty rats divided into five groups (10 animals each) were used in the study—the control group (native muscle; *n* = 10); the ischemia group without C_60_FAS administration (ischemic muscle; *n* = 10); and the group where C_60_FAS was administered once intramuscularly 1 h before muscle ischemia at doses of 0.5 (*n* = 10), 1 (*n* = 10), and 2 mg/kg (*n* = 10), respectively.

It should be emphasized that during the experiments, the control group of ischemic animals received injections of saline with the same dose as C_60_FAS (1 mg/kg; *n* = 10). However, the results obtained did not reveal significant differences in the studied biomechanical and biochemical parameters in this group and in the group of ischemic animals without C_60_FAS administration. It is also important to note that, in accordance with our previous study, the maximum tolerated dose of C_60_FAS was 721 mg/kg for i.p. administration to mice [22].

Anesthesia of the animals was performed by the intraperitoneal administration of nembutal (40 mg/kg). Standard preparation of the experiment also included the cannulation (*a.* carotis communis sinistra) for the therapeutic administration of the drug and pressure measurement, tracheotomy, and laminectomy at the lumbar spinal cord level. For muscle ischemia, the branch of the femoral artery of the animal, which provides blood supply of the experimental muscle, was dragged by ligatures. The duration of ischemia was 3 h. Muscle soleus of the rats were released from the surrounding tissues and their tendons were cut across in a distal part. The ventral roots were cut in places of their exit from the spinal cord for the modulated stimulation of efferents in L4–L5 segments. Filaments of the ventral roots were cut and fixed on stimulating electrodes, and a special device was used for cyclic sequence distribution of electrical signals via the filaments. Stimulation of the efferents was performed by electric impulses with a frequency of 1 to 50 Hz, and the duration of each pulse was 2 ms, formed by using a pulse generator. A control of the external load on the muscle was carried out with the help of an original mechanical stimulator [44]. In the process of analyzing the obtained results, the next parameter was used, namely the integrated muscle power (calculated area under the force curve), which is an indicator of the general performance of the muscle with the applied stimulation pools. The development of the muscle contractile activity was assessed using the method of calculating time intervals when 50% and 25% of the levels of force responses were reached during stimulation.

The level of enzyme content in the blood of the experimental animals (creatinine, LDH, LA, CK, TBARS, H_2_O_2_, GSH, and CAT), as markers of muscle injury [45,46], was determined using clinical diagnostic equipment, namely a haemoanalyzer [15].

Statistical processing of the results was performed using methods of variation statistics using software Original 9.4. At least six repetitions for each measurement were conducted. Data are expressed as the means ± SEM for each group. The differences among the experimental groups were detected by one-way ANOVA followed by Bonferroni’s multiple comparison test. Values of *p* < 0.05 were considered significant.

## 4. Conclusions

Thus, it was shown that the pretraumatic administration of water-soluble C_60_ fullerenes (nanoparticles with size of 0.7–35 nm) at a dose of 1 mg/kg reduces the severity of ischemic damage in the rat muscle soleus by 60–75%. In particular, intramuscular injection of C_60_FAS produces a 52% therapeutic effect at the stages of maintaining maximum force responses during the development of fatigue processes. The preventive effect of C_60_FAS injections on the biomechanical parameters of the transition of ischemic muscle from dentate to smooth tetanus is about 68% of the control values. Finally, the administration of C_60_FAS before muscle ischemia significantly reduced the blood biochemical parameters of the rat (by about 45–60%), which indicates the promise of its use for prophylactic purposes.

## Data Availability

Not applicable.

## References

[1] Erkut B., Özyazıcıoğlu A., Karapolat B.S., Koçoğulları C.U., Keles S., Ateş A., Gundogdu C., Kocaket H. (2007). Effects of ascorbic acid, alpha-tocopherol and allopuri, nol on ischemia-reperfusion injury in rabbit skeletal muscle: An experimental study. Drug Target Insights.

[2] Murdock M., Murdoch M.M. (2012). Compartment Syndrome: A Review of the Literature. Clin. Podiatr. Med. Surg..

[3] Vignaud A., Hourdé C., Medja F., Agbulut O., Butler-Browne G., Ferry A. (2010). Impaired Skeletal Muscle Repair after Ischemia-Reperfusion Injury in Mice. J. Biomed. Biotechnol..

[4] Korthals J.K., Maki T., Gieron M.A. (1985). Nerve and muscle vulnerability to ischemia. J. Neurol. Sci..

[5] Bortolotto S.K., Morrison W.A., Messina A. (2004). The role of mast cells and fibre type in ischaemia reperfusion injury of murine skeletal muscles. J. Inflamm..

[6] Rácz I., Illyés G., Sarkadi L., Hamar J. (1997). The Functional and Morphological Damage of Ischemic Reperfused Skeletal Muscle. Eur. Surg. Res..

[7] Turóczi Z., Arányi P., Lukáts Á., Garbaisz D., Lotz G., Harsanyi L., Szijártó A. (2014). Muscle Fiber Viability, a Novel Method for the Fast Detection of Ischemic Muscle Injury in Rats. PLoS ONE.

[8] Cuzzocrea S., Riley D.P., Caputi A.P., Salvemini D. (2001). Antioxidant therapy: A new pharmacological approach in shock, inflammation, and ischemia/reperfusion injury. Pharmacol. Rev..

[9] Amani H., Habibey R., Hajmiresmail S.J., Latifi S., Pazoki-Toroudi H., Akhavan O. (2017). Antioxidant nanomaterials in advanced diagnoses and treatments of ischemia reperfusion injuries. J. Mater. Chem. B.

[10] Eswaran S.V. (2018). Water Soluble Nanocarbon Materials:A Panacea for All?. Curr. Sci..

[11] Ferreira C.A., Ni D., Rosenkrans Z.T., Cai W. (2018). Scavenging of reactive oxygen and nitrogen species with nanomaterials. Nano Res..

[12] Vereshchaka I.V., Bulgakova N., Maznychenko A., Gonchar O., Prylutskyy Y., Ritter U., Moska W., Tomiak T., Nozdrenko D.M., Mishchenko I.V. (2018). C60 Fullerenes Diminish Muscle Fatigue in Rats Comparable to N-acetylcysteine or β-Alanine. Front. Physiol..

[13] Krusic P.J., Wasserman E., Keizer P.N., Morton J.R., Preston K.F. (1991). Radical Reactions of C60. Science.

[14] Halenova T., Raksha N., Savchuk O., Ostapchenko L., Prylutskyy Y., Ritter U., Scharff P. (2020). Evaluation of the Biocompatibility of Water-Soluble Pristine С60 Fullerenes in Rabbit. BioNanoScience.

[15] Nozdrenko D.M., Zavodovskyi D.O., Matvienko T.Y., Zay S.Y., Bogutska K.I., Prylutskyy Y.I., Ritter U., Scharff P. (2017). C60 Fullerene as Promising Therapeutic Agent for the Prevention and Correction of Skeletal Muscle Functioning at Ischemic Injury. Nanoscale Res. Lett..

[16] Tidball J.G. (2011). Mechanisms of Muscle Injury, Repair, and Regeneration. Compr. Physiol..

[17] Prylutska S., Politenkova S., Afanasieva K., Korolovych V., Bogutska K., Sivolob A., Skivka L., Evstigneev M., Kostjukov V., Prylutskyy Y. (2017). A nanocomplex of C60 fullerene with cisplatin: Design, characterization and toxicity. Beilstein J. Nanotechnol..

[18] Kraemer Â.B., Parfitt G.M., Acosta D.D.S., Bruch G.E., Cordeiro M.F., Marins L., Ventura-Lima J., Monserrat J., Barros D.M. (2018). Fullerene (C60) particle size implications in neurotoxicity following infusion into the hippocampi of Wistar rats. Toxicol. Appl. Pharmacol..

[19] Dryn D.O., Melnyk M.I., Al Kury L.T., Prylutskyy Y., Ritter U., Zholos A.V. (2018). C 60 fullerenes disrupt cellular signalling leading to TRPC4 and TRPC6 channels opening by the activation of muscarinic receptors and G-proteins in small intestinal smooth muscles. Cell. Signal..

[20] Singla R., Sharma C., Shukla A.K., Acharya A. (2019). Toxicity Concerns of Therapeutic Nanomaterials. J. Nanosci. Nanotechnol..

[21] Prilutski Y., Durov S., Yashchuk V., Ogul’Chansky T., Pogorelov V., Astashkin Y., Buzaneva E., Kirghisov Y., Andrievsky G., Scharff P. (1999). Theoretical predictions and experimental studies of self-organized C60 nanoparticles in water solution and on the support. Eur. Phys. J. D.

[22] Prylutska S.V., Grebinyk A.G., Lynchak O.V., Byelinska I.V., Cherepanov V.V., Tauscher E., Matyshevska O.P., Prylutskyy Y.I., Rybalchenko V.K., Ritter U. (2019). In vitro and in vivo toxicity of pristine C60 fullerene aqueous colloid solution. Fuller. Nanotub. Carbon Nanostruct..

[23] Grace P.A. (1994). Ischaemia-reperfusion injury. BJS.

[24] Carvalho A., Hollett P., McKee N. (1995). Recovery of Synergistic Skeletal Muscle Function Following Ischemia. J. Surg. Res..

[25] Kanda K., Hashizume K. (1992). Factors causing difference in force output among motor units in the rat medial gastrocnemius muscle. J. Physiol..

[26] Khoma O., Zavodovs’Kyĭ D., Nozdrenko D., Dolhopolov O., Miroshnychenko M., Motuziuk O. (2014). Dynamics of ischemic skeletal soleus muscle contraction in rats. Fiziol. Zh..

[27] Grottel K., Celichowski J. (1990). Division of motor units in medial gastrocnemius muscle of the rat in the light of variability of their principal properties. Acta Neurobiol. Exp..

[28] Nozdrenko D.M., Abramchuk O.M., Soroca V.M., Miroshnichenko N.S. (2015). Aluminum chloride effect on Ca(2+),Mg(2+)-ATPase activity and dynamic parameters of skeletal muscle contraction. Ukr. Biochem. J..

[29] Pettersson J., Hindorf U., Persson P., Bengtsson T., Malmqvist U., Werkström V., Ekelund M. (2008). Muscular exercise can cause highly pathological liver function tests in healthy men. Br. J. Clin. Pharmacol..

[30] Chamorro Á., Dirnagl U., Urra X., Planas A.M. (2016). Neuroprotection in acute stroke: Targeting excitotoxicity, oxidative and nitrosative stress, and inflammation. Lancet Neurol..

[31] Birben E., Sahiner U.M., Sackesen C., Erzurum S., Kalayci O. (2012). Oxidative Stress and Antioxidant Defense. World Allergy Organ. J..

[32] Bakry R., Vallant R.M., Najam-ul-Haq M., Rainer M., Szabo Z., Huck C.W., Bonn G.K. (2007). Medicinal applications of fullerenes. Int. J. Nanomed..

[33] Ryan J.J., Bateman H.R., Stover A., Gomez G., Norton S.K., Zhao W., Schwartz L.B., Lenk R., Kepley C.L. (2007). Fullerene nanomaterials inhibit the allergic response. J. Immunol..

[34] Srđenović B.U., Slavić M.N., Stankov K.M., Kladar N.V., Jović D.S., Seke M.N., Bogdanović V.V. (2015). Size distribution of fullerenol nanoparticles in cell culture medium and their influence on antioxidative enzymes in Chinese hamster ovary cells. Chem. Ind..

[35] Prylutska S.V., Grynyuk I.I., Matyshevska O.P., Prylutskyy Y.I., Ritter U., Scharff P. (2008). Anti-oxidant Properties of C60Fullerenesin vitro. Fuller. Nanotub. Carbon Nanostruct..

[36] Foley S., Crowley C., Smaihi M., Bonfils C., Erlanger B.F., Seta P., Larroque C. (2002). Cellular localisation of a water-soluble fullerene derivative. Biochem. Biophys. Res. Commun..

[37] Grebinyk A., Prylutska S., Chepurna O., Grebinyk S., Prylutskyy Y., Ritter U., Ohulchanskyy T.Y., Matyshevska O., Dandekar T., Frohme M. (2019). Synergy of Chemo- and Photodynamic Therapies with C60 Fullerene-Doxorubicin Nanocomplex. Nanomaterials.

[38] Halenova T.I., Vareniuk I.M., Roslova N.M., Dzerzhynsky M.E., Savchuk O., Ostapchenko L., Prylutskyy Y.I., Ritter U., Scharff P. (2016). Hepatoprotective effect of orally applied water-soluble pristine C60 fullerene against CCl4-induced acute liver injury in rats. RSC Adv..

[39] Prylutskyy Y.I., Vereshchaka I.V., Maznychenko A.V., Bulgakova N.V., Gonchar O.O., Kyzyma O.A., Ritter U., Scharff P., Tomiak T., Nozdrenko D.M. (2017). C60 fullerene as promising therapeutic agent for correcting and preventing skeletal muscle fatigue. J. Nanobiotechnol..

[40] Kartal H., Küçük A., Kiliçarslan A., Polat Y., Süngü N., Kip G., Arslan M. (2020). The effect of fullerenol C60 on skeletal muscle after lower limb ischemia reperfusion injury in streptozotocin-induced diabetic rats. J. Surg. Med..

[41] Kuznietsova H., Dziubenko N., Hurmach V., Chereschuk I., Motuziuk O., Ogloblya O., Prylutskyy Y. (2020). Water-Soluble Pristine C60 Fullerenes Inhibit Liver Fibrotic Alteration and Prevent Liver Cirrhosis in Rats. Oxidative Med. Cell. Longev..

[42] Prylutskyy Y., Yashchuk V., Kushnir K., Golub A., Kudrenko V., Prylutska S., Grynyuk I., Buzaneva E., Scharff P., Braun T. (2003). Biophysical studies of fullerene-based composite for bio-nanotechnology. Mater. Sci. Eng. C.

[43] Ritter U., Prylutskyy Y., Evstigneev M.P., Davidenko N.A., Cherepanov V.V., Senenko A.I., Marchenko A., Naumovets A.G. (2015). Structural Features of Highly Stable Reproducible C60Fullerene Aqueous Colloid Solution Probed by Various Techniques. Fuller. Nanotub. Carbon Nanostruct..

[44] Nozdrenko D.N., Berehovyi S.M., Nikitina N.S., Stepanova L.I., Beregova T.V., Ostapchenko L.I. (2018). The influence of complex drug cocarnit on the nerve conduction velocity in nerve tibialis of rats with diabetic polyneuropathy. Biomed. Res..

[45] Brancaccio P., Lippi G., Maffulli N. (2010). Biochemical markers of muscular damage. Clin. Chem. Lab. Med..

[46] Gonchar O.O., Maznychenko A.V., Bulgakova N.V., Vereshchaka I.V., Tomiak T., Ritter U., Prylutskyy Y.I., Mankovska I.M., Kostyukov A.I. (2018). C60 Fullerene Prevents Restraint Stress-Induced Oxidative Disorders in Rat Tissues: Possible Involvement of the Nrf2/ARE-Antioxidant Pathway. Oxidative Med. Cell. Longev..

